# A Review of Joining Technologies for SiC Matrix Composites

**DOI:** 10.3390/ma18092046

**Published:** 2025-04-30

**Authors:** Yongheng Lu, Jinzhuo Zhang, Guoquan Li, Zaihong Wang, Jing Wu, Chong Wei

**Affiliations:** 1 Nuclear Fuel Components Key Laboratory of Inner Mongolia Autonomous Region, China North Nuclear Fuel Co., Ltd. (CNNC), Baotou 014035, China; yonghenglu2025@163.com (Y.L.); zaihongwang@163.com (Z.W.); 2 School of Mechanics and Civil Engineering, Northwestern Polytechnical University, Xi’an 710072, China; 18292686619@163.com (J.Z.); guoquanli2025@163.com (G.L.); rayselwu@nwpu.edu.cn (J.W.); 3 Research & Development Institute, Northwestern Polytechnical University in Shenzhen, Shenzhen 518057, China

**Keywords:** SiC ceramics, SiCf/SiC composites, bonding technology, joint, base material, interface

## Abstract

SiC matrix composites are widely used in high-temperature structural components of aircraft engines and nuclear reactor materials because of their excellent properties such as their high modulus, high strength, corrosion resistance, and high-temperature resistance. However, the bonding of SiCf/SiC composites poses significant challenges in practical engineering applications, primarily due to residual stresses, anisotropy in composite properties, and the demanding conditions required for high-performance joints. This work reviews various bonding technologies for SiC ceramics and SiC matrix composites. These include solid-state diffusion bonding, NITE phase bonding, direct bonding without filling materials, MAX phase bonding, glass ceramic bonding, polymer precursor bonding, metal brazing bonding, and Si-C reaction bonding. Key results, such as the highest bending strength of 439 MPa achieved with Si-C reaction bonding, are compared alongside the microstructural characteristics of different joints. Additionally, critical factors for successful bonding, such as physical mismatch and metallurgical incompatibility, are discussed in detail. Future research directions are proposed, emphasizing the optimization of bonding techniques and evaluation of joint performance in harsh environments. This review provides valuable insights into advancing bonding technologies for SiC composites in aerospace and nuclear applications.

## 1. Introduction

Silicon carbide (SiC) materials have gained prominence in the aerospace, nuclear, semiconductor-device, heat-exchanger, and other high-performance industries due to their exceptional thermal stability, corrosion resistance, high-temperature resistance, radiation resistance, and mechanical robustness [1,2]. In particular, SiCf/SiC composites provide better fracture toughness [3] and thermal shock resistance [4] than SiC ceramic materials while maintaining the excellent properties of SiC ceramic materials [2,5]. In recent years, SiCf/SiC composites have been widely used in high-temperature structural components of aero-engines and nuclear reactor materials [6]. However, their widespread implementation faces significant challenges stemming from inherent limitations in machinability and the technical complexity of manufacturing intricate geometries. This predicament has elevated the strategic importance of joining technologies in realizing the full potential of SiC-based components [7].

The highly covalent nature of SiC makes it challenging to join SiCf/SiC composites by fusion welding. The MAX phases constitute a class of laminated materials composed of an early transition-metal (M), an A-group element (A), and C, N, B, and/or P (X). The current bonding techniques mainly contain solid-state diffusion bonding [8], NITE phase bonding [9,10], direct bonding without filling materials [11], MAX phase bonding [12,13], glass ceramic bonding [14], polymer precursor bonding [15], metal brazing bonding [16], and Si-C reaction bonding [17]. These bonding techniques can be further categorized into two major groups: pressure bonding and pressureless bonding, in which solid-state diffusion bonding, NITE phase bonding, direct bonding without filling materials, and MAX phase bonding require pressure to make the bonding during the bonding process and the rest of the bonding methods can be carried out under pressureless conditions. Among them, the bonding joints prepared by pressure bonding tend to have higher densities and excellent mechanical properties in the joints. Pressureless bonding does not cause damage to the base material because no pressure is applied. Especially for the encapsulated bonding of the end plugs of nuclear fuel cladding, it is not possible to encapsulate the joints using pressurization because they cannot withstand excessive pressure [18]. Among these bonding techniques, except for the direct bonding technique without bonding layers, the other bonding methods require using non-SiC-based or SiC-based materials as fillers for their bonding. Due to the mismatch of the coefficient of thermal expansion (CET) between the filler and the base material [11], there are often thermally induced residual stresses in the joints that make the joints perform worse than the base material. Usually, when selecting fillers, we choose the coefficient of thermal expansion and other thermophysical properties that are the same as or close to those of the base material to overcome the heat-induced residual stresses in the joints and obtain good-performance joints. Different bonding techniques offer unique advantages for various applications. Direct bonding without filling materials eliminates intermediary layers, preserving material properties and ensuring purity, making it ideal for fields like electronics and aerospace. Metal solid-state diffusion bonding creates strong, durable bonds without melting the metals, providing excellent mechanical strength and resistance to fatigue, which is beneficial in the aerospace, automotive, and nuclear industries. The fatigue resistance of Cf/SiC composites has been a subject of extensive research. For instance, a study by [19] investigated the fatigue behavior of these composites under various loading conditions, providing valuable insights into their performance. NITE phase bonding utilizes nano-sized particles to form a new phase, enhancing bond strength without compromising material properties, making it ideal for advanced ceramics in aerospace, electronics, and defense applications. A recent review by Zhang et al. [20] has provided a comprehensive overview of joining methods for Cf/SiC ceramic matrix composites. Each technique offers specialized benefits for high-performance materials and applications, but there is still a lack of evaluation of the performance of these joints in practical applications. Zhang et al. [20] also mentioned evaluation methods such as scratch testing, which is critical for assessing surface properties and degradation mechanisms in wear or abrasive environments. Future studies may incorporate scratch testing to further assess joint durability under abrasive conditions. However, most of the conducted studies have only analyzed and characterized the joints’ mechanical properties, micro-morphology, and formation mechanism, and we all know these are insufficient to provide data support for the joints in practical applications.

This work mainly introduces the different bonding technologies that can be applied to SiCf/SiC composites and describes their basic mechanisms, microstructures, joint strength, performance evaluation, and advantages and disadvantages. In addition, the corresponding suggestions and thoughts are put forward for each bonding technology. Finally, the development trend of SiCf/SiC composite bonding technology is prospected.

## 2. Bonding Technology of SiCf/SiC Composites

There are two significant challenges in the bonding of SiC_f_/SiC composites: firstly, the question of whether the material can be successfully bonded, and secondly, whether the bonding joints can withstand service requirements in harsh environments. In detail, the successful bonding of SiC_f_/SiC composites primarily encounters issues related to physical mismatch and metallurgical incompatibility [21]. Most of the bonding methods require filler materials to bond the joints, and the filler materials are often very different from the base material in properties, such as in metal-base brazing bonding [18,22]. There are significant differences in chemical bonds and thermal physical properties between the filler materials and the base materials. Even if the filler materials are not used, or the properties of the filler materials are close to those of the base material, the bonding method often requires a series of harsh bonding conditions such as high temperature and high pressure. SiC has a highly stable electronic coordination structure. As a result, filler materials generally exhibit poor or even no wettability on its surface, making it difficult to form a strong interfacial reaction and establish chemical bonding. In addition, it is easy to produce various hard and brittle compounds at the bonding interface, resulting in poor performance at the bonding joint.

The development of SiC_f_/SiC composite bonding technology is not only solely aimed at establishing connections but also focused on ensuring that these joints can endure diverse complex and harsh environments. The main problem faced by various current bonding technologies is that the shear strength of the joint has different degrees of deviation and the instability of the strength deviation limits the engineering application of the joint in practice. In addition, there is a lack of evaluation of joint performance in most bonding studies.

### 2.1. Bonding Without Filling Materials

The technology of bonding without filling materials refers to the direct bonding of two materials (homogeneous materials or dissimilar materials) without adding any filling materials, and the bonding is realized through two bonding mechanisms: interface reaction and element diffusion. The advantage of this bonding technology is that for the bonding of SiC_f_/SiC composites, the residual stress caused by the mismatch of thermal expansion coefficient will not be generated in the joint of the homogeneous material, and the bonding layer material is mainly SiC. However, for the bonding between SiC composites and metals, there is no filling material to relieve the residual stress caused by heat in the joint because it is directly bonded with dissimilar materials, resulting in poor mechanical properties for the joint [23]. The quantitative analysis of residual stress involves various techniques such as X-ray diffraction (XRD), neutron diffraction, Raman spectroscopy, and finite element analysis (FEA) to measure and understand the distribution, magnitude, and effects of internal stress in materials or structures. However, the quantitative analysis of residual stress at the bonding interface remains a challenging issue for researchers. Exploring suitable methods for interface residual stress analysis in the future will facilitate the development of optimized bonding processes. To mitigate residual stress at the bonding interface, appropriate heat treatment processes or bonding materials with matching thermal expansion coefficients [24,25,26,27,28] are typically used to redistribute or relieve residual stress. This approach can further enhance the strength, durability, and fatigue resistance of the joint, ensuring the reliability and longevity of engineering components. Because no filling material is used, the whole bonding process often requires a high temperature and pressure. High-pressure conditions can reduce the gap between the materials and shorten the diffusion distance of the elements; only in this way can the interface reaction and element diffusion in the bonding joint be promoted, the bonding time be shortened, and the bonding temperature be reduced. Therefore, the surface roughness of the bonding material often needs to be very small, and a large surface roughness will make the mechanical properties of the bonding joint poor [24]. A smooth surface is more conducive to the diffusion of atoms in the bonding joint. Therefore, when SiC_f_/SiC composites are bonded, Chemical Vapor Infiltration (CVI) technology is often required to treat the surface to obtain a surface with more minor roughness.

**Figure 1:**
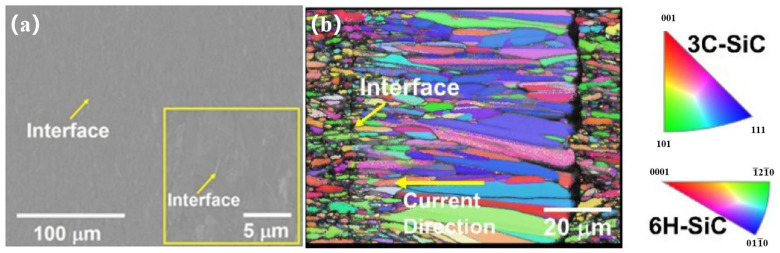
Microstructure of SiC direct bonding joint at 2160 °C for 1 min using Electric-Current-Assisted Bonding (ECAJ) technology. (**a**) SEM image; (**b**) EBSD analysis [26].

With the development of Spark Plasma Sintering (SPS) technology, it is now easier for most researchers to realize the bonding without filling materials by using this technology [11,25,26]. This technology is a new method of ceramic sintering densification, which can couple an electric field, temperature field, and force field to realize the application of pulsed current to the graphite mold, thus generating the Joule thermal effect, which increases the temperature rapidly. At the same time, the current promotes the rapid sintering of powder and realizes the controllable structure of the material. This is considered a low-temperature, fast, and efficient material preparation method. This bonding technology can greatly reduce the bonding temperature and shorten the holding time, thus greatly reducing the damage to the SiC substrate during the bonding process. When using filler materials to bond SiC_f_/SiC composites, the performance of most filler materials is always lower than that of the base materials, which is also the main reason for the failure of most bonding joints, and the direct bonding of SiC_f_/SiC composites can avoid problems such as the failure of bonding joints caused by filler materials. Li et al. [26] successfully obtained the direct bonding of SiC_f_/SiC composites through Electric-Current-Assisted Bonding (ECAJ) technology. The rapid growth of textured silicon carbide crystals was observed at the interface (Figure 1b) while a nearly defect-free bonding joint was successfully formed in the adjacent region (Figure 1a). Aroshas et al. [27] successfully connected SiC with Spark Plasma Sintering (SPS) and obtained an almost seamless joint. Nano-indentation was used to test the interface of the joint and the base materials, and similar mechanical properties were obtained.

**Figure 2:**
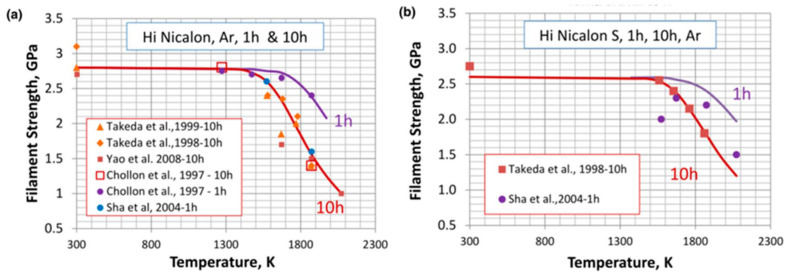
The strength loss with temperature in an Ar gas environment for two types of fibers, (**a**) Hi Nicalon [32,33,34,35,36] and (**b**) Hi Nicalon S [30,33,36].

In addition, SiC materials can also be directly bonded through traditional hot pressing methods. Matsuo et al. [28] and Naka et al. [29] used hot pressing methods to bond SiC with W and SiC with Nb, where the shear strength of the joint between SiC and Nb was 108 MPa. However, such a bond often requires a long heat preservation time to diffuse atoms. For SiCf/SiC composites, grain growth in such a bonding environment will lead to degradation and strength loss for SiC fibers, as shown in Figure 2, which may lead to the degradation of the service performance of the joint [30]. Zhou et al. [11] applied a direct current electric field to both ends of a SiC base material in the air to achieve the ultra-low temperature bonding of SiC at 400 °C, and the joint mainly depended on SiO_2_ at the interface. Even if low-temperature bonding technology is developed, direct bonding without filling materials is still difficult for SiC_f_/SiC composites, and the surface of SiC_f_/SiC composites is difficult to smooth. However, a layer of SiC can be deposited on the surface of SiC_f_/SiC composites by Chemical Vapor Deposition (CVD) to reduce surface roughness [31].

Table 1 summarizes the relevant data on bonding without filling materials. The process often requires demanding bonding conditions (minor surface roughness, high bonding temperature, and high bonding pressure), significantly limiting these materials’ engineering applications. At present, the technology of bonding without filling materials is more about the bonding of SiC ceramics because the surface is smoother, but for SiC_f_/SiC composites, the temperature that the fiber can withstand is generally not more than 1600 °C, so bonding at high temperatures may damage the base materials. With the continuous progression of fiber preparation technology, the production of fibers resistant to high-temperature environments will promote the development of bonding technology. Most existing technologies of bonding without filling materials aim to promote the high diffusion rates of atoms in the base materials so that the SiC ceramics can grow rapidly and finally be successfully bonded. Whether new technologies can be developed to promote the rapid and efficient diffusion of atoms in the future is also the key to the technology of bonding without filling materials.

### 2.2. Metal Solid-State Diffusion Bonding

Metal solid diffusion bonding refers to the metal filler added to the base material for bonding. The whole bonding process of the filler only exists in the form of a solid phase under a high-temperature and -pressure environment. The base material SiC and the metal surface contact each other, and atomic diffusion and chemical reactions occur at the interface, finally generating a dense diffusion layer and reaction layer. In comparison to direct bonding without an interlayer, the use of a filler facilitates bonding due to its higher diffusion rate compared to Si and C in SiCf/SiC composites. Nevertheless, as the bonding process occurs entirely in the solid state, it depends exclusively on atomic diffusion. The bonding process requires high temperature and pressure to promote diffusion. If the joint is bonded under vacuum and no pressure, the strength of the joint is often low, there are many gaps, and the degree of densification of the joint is not high [39], which is mainly caused by insufficient diffusion. However, the metal solid diffusion joint has higher strength under the reaction of high temperature and pressure. Zhao et al. [40] bonded C_f_/SiC by Spark Plasma Sintering (SPS) technology at 1600 °C and 50 MPa and the shear strength of the joint was 61 ± 6 MPa. To avoid the oxidation of SiC_f_/SiC composites and fillers, vacuum or inert gas was usually used in the bonding process [25,41,42]. In actual bonding, the filler is generally a powder substance or a metal foil, but the metal foil is not conducive to the bonding of complex structures in practical engineering applications. Fillers usually comprise alloys or pure metals containing active metal elements such as Al, Zr, Nb, Mo, Ta, Mn, Cr, V, and Ti. Active metal elements can be diffused with Si and C in SiC to generate silicide (MSi_y_) or carbide (MC_x_). The equation is as follows [41]:

$$\left(xy\right)SiC+\left(x+y\right)M\to \left(y\right)MC_{x}+\left(x\right)MSi_{y}$$

The composition and physical properties of metal solid diffusion interface products are highly correlated with the choice of bonding layer material, bonding temperature, and holding time. Therefore, the effective control of the interface reaction is the key factor to achieving a high-performance joint.

**Figure 3:**
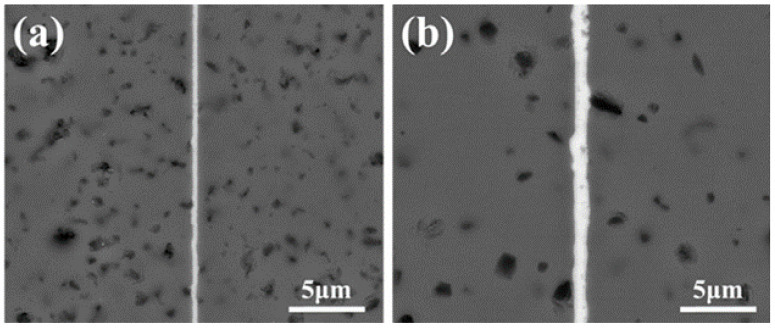
Microstructure of SiCf/SiC composites bonded by Ti interlayer with different thicknesses: (**a**) a 100 nm Ti interlayer; (**b**) a 500 nm Ti interlayer [44].

Martinelli et al. [43] took Mo as the bonding layer and held it at 1200–1500 °C for 15–120 min for hot pressing treatment. They compared the effects of different bonding temperatures and holding times on SiC-Mo joints and concluded that the optimal joints could be obtained at 1400 °C for 1 h, with an average shear strength of 50 MPa. The wrong choice of bonding layer and bonding process often causes joints to produce residual stress caused by heat, resulting in joints cracking. The thickness of the bonding layer also has a particular influence on the performance of a joint. Yang et al. [44] took Ti as the bonding layer, controlled the bonding layer thickness by physical vapor deposition, and obtained SiC-Ti joints by Electric-Current-Assisted sintering technology. In addition, the effect of bonding layer thickness on the joint performance is investigated. It is concluded that the thickness of the Ti bonding layer under the same heat treatment condition will affect the concentration and distribution of Si and C reactants in the resulting bonding layer and then the bonding layer with different phase composition and phase distribution will have an impact on the performance of the joint. Figure 3 shows the microstructure of the joint with different thicknesses bonding layers. When a 100 nm Ti interlayer is employed (Figure 3a), the detrimental brittle Ti_5_Si_3_ phase is largely suppressed by a continuous, dense TiC layer at the interface. And the sample with a 500 nm Ti interlayer forms Ti_3_SiC_2_ in Figure 3b, achieving the highest strength of 205.7 MPa among the various Ti coating thicknesses.

Kishimoto et al. [45] tested the diffusion joint under irradiation in a pressurized water flow environment. The joint did not exhibit significant damage, indicating that diffusion bonding technology demonstrates a certain degree of irradiation resistance. Table 2 summarizes the bonding procedures and mechanical property evaluations of metal-solid-diffusion-bonded SiC and SiC matrix composites in recent years. Future development of this bonding technology aims to enhance diffusion rates, shorten bonding time, lower the required bonding pressure, and minimize damage to the base materials during the process.

### 2.3. NITE Phase Bonding

**Figure 4:**
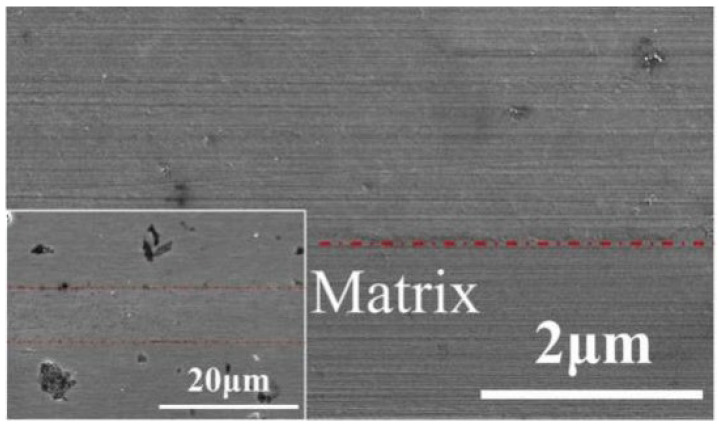
Microscopic morphology of NITE-phase-prepared bonding joints [9].

Nano-infiltration and Transient Eutectic (NITE) bonding technology is a method to bond the base material by the liquid phase sintering of nano SiC power under the action of sintering aid. A sintering aid can reduce the sintering temperature of SiC to achieve the low-temperature densification of the SiC bonding layer. The choice of sintering aid and the amount of sintering aid are very important to the densification degree and joint strength of the bonding layer. Bonding thickness is also a key factor affecting the quality of the NITE welding layer. Especially when bonding SiC_f_/SiC composites, CVI treatment on the bonding surface can improve the wettability of the filler material on the base material surface and ultimately make the SiC generated by the bonding layer denser and the joint strength higher. The bonding layer prepared by NITE phase bonding is SiC, so it has good compatibility with the base material and the joint has high strength. As shown in Figure 4, Zhan et al. [9] prepared a joint without defects at 1650 °C and 30 MPa using Al_2_O_3_-Y_2_O_3_-MgO-CaO as a sintering aid. An average shear strength of 69.5 ± 8.9 MPa was obtained.

A high temperature and pressure are often required in the bonding process to promote the sintering densification of the bonding-layer SiC. The bonding temperature is usually higher than 1500 °C, and the pressure is greater than 10 MPa, but the base material may be damaged in this process. Jung et al. [56] used mixed Al_2_O_3_, Y_2_O_3_, SiO_2_, and SiC powders to bond SiC. The bonding temperature was ranging from 1500 °C to 1900 °C, and the bonding pressure was ranging from 5 MPa to 20 MPa for 1 h. Finally, it has been found that the bonding strength of the joint increases with increases in the bonding temperature and pressure, but a high bonding temperature and pressure will cause the deformation of SiC.

**Figure 5:**
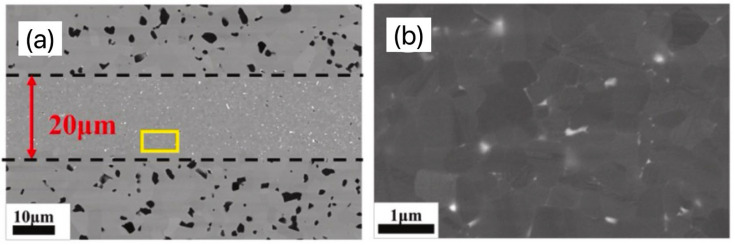
Microscopic morphology diagram of pressureless NITE bonding joint [10]. The microstructure of the joints at 1750 °C for different holding times: (**a**) 2.0 h, (**b**) the magnified image of the yellow rectangle in (**a**).

**Figure 6:**
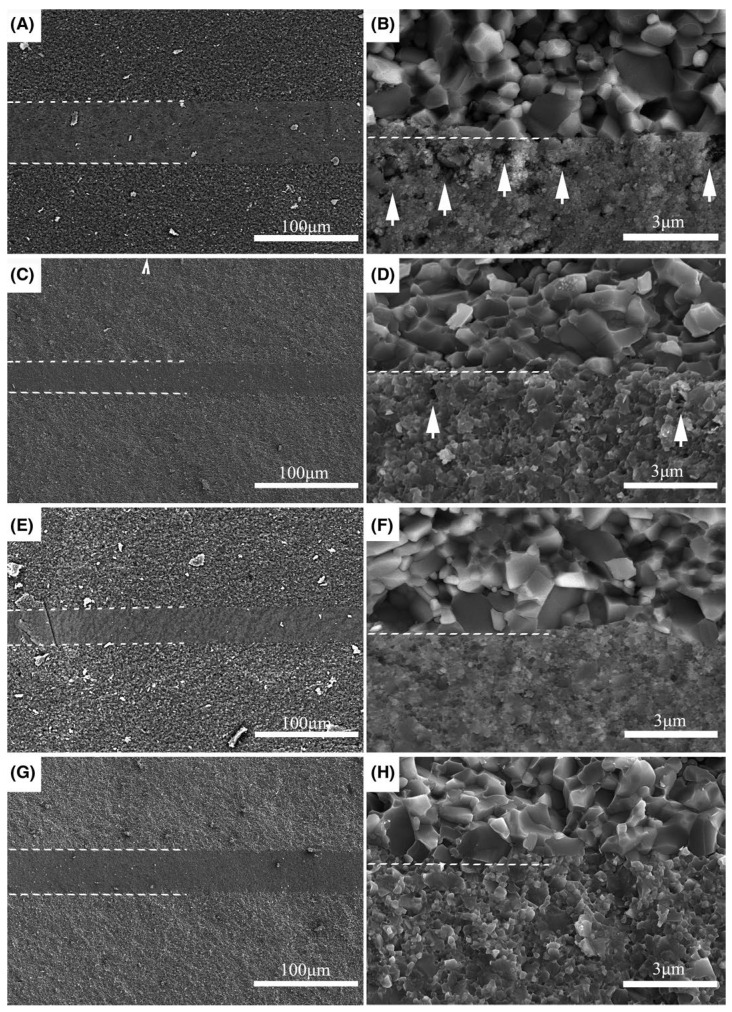
SEM images of NITE bonding joints under different temperatures for 10 min under the pressure of 20 MPa: 1500 °C (**A**,**B**), 1600 °C (**C**,**D**), 1700 °C (**E**,**F**), and 1800 °C (**G**,**H**) [57].

Table 3 summarizes the bonding process and mechanical property evaluation of NITE-bonding SiC and SiC matrix composites in recent years. NITE bonding is a bonding technology newly developed in recent years, and its high temperature and pressure bonding conditions make it impossible to bond some thin, brittle structures that cannot withstand more significant pressure. However, Zhou et al. [10] used AlN-Y_2_O_3_ as a sintering aid to hold heat for 2 h at 1750 °C. During the bonding process, they only relied on graphite fixtures to complete the bonding in a nearly pressureless way. As shown in Figure 5, the bonding layer was dense without defects and the joint strength was high. The future development trend of NITE bonding will be to achieve the densification of the joint by a pressureless method. Since SiC is used as the bonding layer, the joint usually has excellent mechanical properties at high temperatures. Zhou et al. conducted a high temperature bending test on the NITE joint prepared by them, and it still had a bending strength of more than 150 MPa at 1400 °C. Niu et al. [57] used Spark Plasma Sintering (SPS) at a bonding temperature range of 1500–1800 °C to join silicon carbide (SiC) ceramics using NITE bonding and Al_2_O_3_-Ho_2_O_3_ as bonding adhesives. Figure 6 presents SEM images of a joint at different bonding temperatures. At bonding temperatures of 1500 °C and 1600 °C, the interlayer is evenly distributed (Figure 6A,C). However, as indicated by the arrows in the high-magnification images (Figure 6B,D), the interlayer is not fully densified, and the presence of pores is observed, resulting in shear strengths of 53.9 MPa and 78.4 MPa, respectively. As the bonding temperature increases to 1700 °C, the joint becomes fully densified (Figure 6E,F), achieving a high shear strength of 157.8 MPa. Similarly, at 1800 °C, the joint remains densified (Figure 6G,H), with noticeable grain growth in the interlayer, while the shear strength remains at 155.2 MPa. Therefore, increasing the bonding temperature promotes joint densification, which, in turn, enhances the bonding strength. NITE bonding has a high application value in the field of nuclear energy. Through experiments, Koyanagi et al. [58] verified the stability of NITE-sintered SiC technology under irradiation. However, we must still perform an irradiation assessment of the NITE joint to determine its irradiation stability. In some studies [10,59], various corrosion tests (hydrothermal corrosion, acid corrosion) were carried out on SiC prepared by NITE, which all performed excellently. We may refer to the performance assessment of joints.

### 2.4. MAX Phase Bonding

The MAX phase is a new class of ternary layered compounds composed of M, A, and X elements. Its general chemical formula is M_n+1_AX_n_, where M is a pre-transition metal element, A is a group A element, and X is carbon or nitrogen. MAX phases combine the advantages of metals (such as electrical and thermal conductivity, and ease of mechanical processing) and ceramics (such as high modulus, and resistance to high temperatures, oxidation, and corrosion).

The aim of MAX phase bonding is to bond SiC_f_/SiC composites under specific temperature and pressure conditions by using a MAX phase material as the bonding layer. With the continuous development of bonding technology, the MAX phase has been widely used as an antioxidant filler for high-temperature structural parts and ceramic bonding. At present, Ti_3_SiC_2_ and Ti_3_AlC_2_ are the most common MAX phase materials and are regarded to be suitable fillers for bonding SiC matrix composites.

Fitriani et al. [13] used thin Ti_3_AlC_2_ fillers to bond SiC materials and bonded them for 5 or 8 h under 1900 °C and 3.5 MPa pressure, and the joint strength was up to 300 MPa. In the bonding process, Ti3AlC2 decomposes into TiC, Al, and Ti, and a high temperature leads to the evaporation of Al and Ti, leaving some small holes. The TiC generated during the reaction diffuses into the bonding material through solid-state diffusion, effectively modifying the interface. Additionally, precipitation hardening improves the mechanical properties of the bonding layer, thereby reducing its adverse impact on the base material.

Table 4 summarizes the bonding process and mechanical property evaluation of SiC and SiC matrix composites using the MAX phase as a bonding layer in recent years. The MAX phase is widely used because it has advantages in both metals and ceramics. However, as a bonding filler material, high temperature and pressure are often required in the bonding process due to its ceramic properties. In addition, the MAX phase will cause its decomposition at a specific temperature, which is also a problem that we should consider when using the MAX phase. MAX phases exhibit excellent irradiation resistance; however, limited research has been conducted on the irradiation behavior of joints prepared using MAX phases. Further evaluation of their environmental performance is needed to support their broader application.

### 2.5. Glass Ceramic Bonding

**Figure 7:**
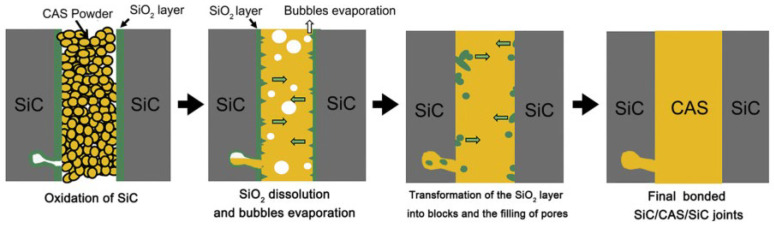
Bonding mechanism of glass ceramics [64].

Glass-ceramic bonding aims to heat a glass ceramic so that the glass ceramic and the surface of the material that needs to be bonded achieve good penetration, and are finally tightly bonded together, then cools the glass ceramic and bonding materials to room temperature; glass ceramics can still be firmly combined with the required bonding materials. The mechanism of glass ceramic bonding is generally divided into four processes [64], as shown in Figure 7: the (1) oxidation of the SiC matrix, (2) dissolution and evaporation of SiO_2_, (3) transformation of SiO_2_ into bulk and pore filling, and (4) formation of uniform and dense SiC/glass ceramic/SiC joints.

The bonding of SiC matrix composites by glass ceramics can be carried out in air and does not require pressure. The wettability and complexity of glass ceramics and SiC matrix composites are also excellent, and the bonding cost is low. The problem that needs to be solved and explored is how to correctly and reasonably design the type and composition of glass solder to achieve good chemical compatibility and a thermal expansion coefficient for glass ceramics and bonding materials. On the other hand, the water–oxygen corrosion resistance of glass ceramics is weak, so improving the water–oxygen corrosion resistance is also a big problem that needs to be solved.

In Table 5, the bonding process and performance evaluation of glass-ceramic-bonded SiC and SiC matrix composites in recent years are summarized. Glass ceramic bonding joints usually have high strength, but the moderate softening temperature of the glass phase makes them often unsuitable for high-temperature applications. The advantage of glass ceramic bonding is that the coefficient of thermal expansion of the bonding layer can be regulated, and the coefficient of thermal expansion of the filling material can usually be regulated to be close to the base material to reduce the thermal mismatch generated during the bonding process.

### 2.6. Polymer Precursor Bonding

Polymer precursor bonding is the use of silicone polymer fillers, such as polycarbosilane, polysiloxane, or polysiloxane, in the bonding process through cross-linking curing into thermosetting polymer and then through high-temperature cracking to obtain ceramic materials finally. The microstructure and phase at the bonding interface can be controlled by controlling the molecular weight and composition of the precursor silicone polymer and the heat treatment conditions.

Colombo et al. [73] prepared the SiC_f_/SiC composite material joint using a ceramic precursor polymer. The polymer was transformed into a ceramic material during pyrolysis at high temperatures and bonded with the composite material to form an adhesive. Therefore, active or inert fillers were mixed with the preceramic polymer to reduce the volume shrinkage during pyrolysis.

In particular, the joint achieved using a silicone resin with Al-Si powder as a reactive additive showed a high shear strength (maximum 31.6 MPa). Yuan et al. [74] used polysiloxane silicone resin (YR 3184, GE Toshiba Silicones) as a bonding material to prepare reaction-sintered silicon carbide (RBSiC) joints and pressureless sintered silicon carbide (SiC) joints and heat-treated them with nitrogen at 1200 °C. The maximum three-point bending strength of a reaction-sintered silicon carbide ceramic joint is 197 MPa. The maximum three-point bending strength of a pressureless sintered silicon carbide joint is 163 MPa. Tang et al. [75] prepared a room-temperature curing heat-resistant adhesive with a wide operating temperature range through organic and inorganic modification. In the temperature range of 400 °C~600 °C, the bonding strength of the ceramic precursor polymethyl siloxane and silicon carbide is low due to the decomposition of the polymer network. Therefore, epoxy resin modification is used to decompose it at a high temperature above 500 °C to form a solid blending copolymerization network. Active filters and ceramic precursors improve the bond strength with an increasing temperature, thus eliminating the weak bond stage of 400 °C~600 °C. Modifying the filler significantly improves its bond strength at high temperatures above 1000 °C. The results in the aforementioned study showed that after heat treatment at 25 °C~1500 °C, the bonding strength of the modified adhesive was 9.29 ± 0.56 MPa~37.28 ± 1.33 MPa at room temperature and 8.21 ± 0.40 MPa at 25 °C~800 °C. The adhesive has a broad application prospect in engineering ceramic bonding.

In addition, Zhao et al. [15] used polycarbosilane (PCS) as the bonding material. They adopted a screen-printing method to distribute PCS on the surface of SiC ceramics evenly and realized the pressureless bonding of SiC ceramics at a low temperature of 1500 °C. X-ray diffraction analysis shows that the pyrolysis product of PCS is single-phase SiC. The middle layer thickness of the SiC joint is about 2 μm. This ultra-thin interlayer reduces the possibility of defects and has an average shear strength of 105.8 ± 10.4 MPa. The printing method using PCS as a bonding material has the advantages of simple processes, low costs, high bonding strength, and reasonable practicability in SiC ceramic bonding. Tang et al. [76] proposed a new two-step bonding technique for preparing in situ SiC nanowire-reinforced SiCf/SiC joints. Scanning electron microscopy (SEM) was used to observe the microstructure of the bonding layer after pyrolysis and the bonding joint after the C–Si reaction. SEM images showed that the SiC nanowires generated inside the joining layer displayed a needle-like morphology, at the tip of which were small pellets, and EDS results showed that the middles of SiC nanowires and tips of nanowires were composed of C and Si, and C, Si, and Fe, respectively. The ratio of C:Si confirmed that stoichiometric SiC nanowires had formed in the stage of pyrolysis of the joining layer. Although EDS provides only semiquantitative elemental analysis, the measured elemental distributions and relative compositions were consistent with the expected stoichiometry of the reaction products within the technique’s detection limits [77]. Meanwhile, the generation of the gas phase and the shrinkage of volume in the pyrolysis process led to the formation of pores at the interface, affecting the joint’s air tightness and strength. In addition, cracks can also form when the stress caused by the change in a polymer–ceramic structure is not sufficiently released. The precursor filler needs to be used carefully to obtain a good joint as it is challenging to convert the polymer directly to dense SiC. However, repeated infiltration treatment after bonding can significantly improve the bonding strength of the joint. For example, in a study [1], after three cycles of infiltration, the joint strength of a SiC_f_/SiC joint with polysiloxane as the intermediate layer was increased from 107.3 MPa to 169.1 MPa, and the joint strength was further increased to 177 and 256.1 MPa after the addition of SiC and Ni nanoparticles.

The polymer precursor bonds to form SiC matrix materials at an interface by pyrolysis at 1200~1800 °C. Due to the porosity at the interface, only a moderate strength of about 100 MPa can be obtained, which is caused by the mass loss and volume change during the conversion of the polymer to the ceramic. In addition, due to the inherent instability of silicon carbide oxide, SiC joints produced by ceramic precursors are usually unstable in radiation environments. Therefore, to improve the suitability of nuclear reactors, the precursor is expected to be converted into highly crystalline SiC. Table 6 shows the bonding process and mechanical property evaluation of polymer-precursor-bonded SiC and SiC matrix composites.

### 2.7. Metal-Brazed Bonding

Metal brazing is the most widely used and mature bonding method. Metal brazing bonding refers to using a solder containing metal elements. The melting point of the metal solder is generally lower than that of the material to be bonded, the metal solder is heated to the melting temperature (the temperature needs to be lower than the melting point of the base metal), the liquid solder is used to moisten and fill the gap of the solid base materials, and the material is bonded by diffusion and chemical reaction. The metal fillers used in the bonding process of SiC_f_/SiC composites generally contain Al, Zr, Nb, Mo, Ta, Mn, Cr, V, Ti, and other active elements, and such active metal elements generally have more excellent wettability for SiC ceramics and SiC matrix composites in the brazing process. They can react with carbon to form carbides. Finally, the material is bonded by an interfacial reaction. In addition to adding active metal elements to the filler metal, some substances with a specific solubility to carbon can also be used, such as Fe, Co, and Ni. Although these metal elements cannot react with carbon, they can dissolve a certain amount of carbon and thus can form a solid solution. To avoid the oxidation of the filler metal during bonding, providing a good vacuum environment or a protective atmosphere is necessary. Metal brazing bonding has the advantages of pressureless bonding, low process costs, a simple process technology, high joint strength, and an adjustable joint structure. However, the thermal expansion coefficient of the metal brazing filler metal is generally different from that of the base metal, and the mechanical properties of base metal after bonding are often poor because of residual stress caused by heat.

Shi et al. [79] used Ni-based alloys to bond SiC matrix composites. During the bonding process, the bonding layer would produce graphite, which would deteriorate the mechanical properties of the bonding layer. By adding the Zr element to the fillers layer, the phase composition of the bonding layer could be changed to reduce the generation of graphite. When the Zr content was increased to more than 40%, the brazed weld would no longer produce graphite. The experiment significantly improved the radiation resistance of the material through the Zr element and increased the shear strength of the bonding layer by three times. Valenza et al. [80] used an Al-Ti metal filler metal to bond SiC to SiC to improve the performance of metal-brazed joints. They formed a Ti_3_Si (Al)C_2_ phase structure in situ, which effectively improved the mechanical properties of the joint. The shear strength of the joint prepared by the osmotic bonding of the filler metal could reach 296 MPa. This bonding method to prepare the MAX phase in the joint was different from the previous pressure connection to prepare the MAX phase and had a particular innovation.

In addition, metal brazing bonding because of the use of metal fillers can bond the SiC base material and the SiC base material with the metal material. Lin et al. [81] believe that achieving the bonding of ceramics and metals is of great significance for expanding its application in the nuclear industry. They used a Nb0.74CoCrFeNi2 eutectic high-entropy alloy filler to conduct brazing bonding experiments on SiC and Mo. They studied the microstructure and mechanical properties of a joint at different brazing temperatures. With an increase in temperature, the eutectic structure gradually disappeared and the lamellar structure formed at the brazed joint. The increase in the MoNi phase content could improve the joint strength due to the difference caused by the coefficient of thermal expansion and the lattice mismatch between the phases. The joint exhibited a maximum shear strength of 62 MPa at 1300 °C. Meanwhile, the finite element analysis results showed that the presence of Cr_0.46_Mo_0.4_Si_0.14_ phase led to the concentration of residual stress, and the brittle fracture mainly occurred in the Cr_0.46_Mo_0.4_Si_0.14_ phase, which led to the fracture of the joint. This study provided a new research direction using eutectic high-entropy alloys as fillers to establish a reliable bond between ceramics and metals.

For the application of metal brazing bonding for nuclear reactors, elements with excellent radiation resistance should be selected, and the oxidation resistance of the joint should be considered. In recent years, the widely used metal–silicon alloy system has been used to bond SiC matrix materials. The addition of Si improves the oxidation resistance of the joint to a certain extent, but the presence of free silicon in the joint shows anisotropic expansion under neutron irradiation, thus reducing the strength of the joint [82]. Table 7 summarizes the bonding process and mechanical property evaluation of metal-brazed SiC and SiC matrix composites.

### 2.8. Si-C Reaction Bonding

Si-C reaction bonding is based on the reactive sintering method for preparing SiC ceramics. During this process, a prefabricated carbide is first placed on the surfaces to be bonded and solidified into a skeleton at a low temperature. Subsequently, molten Si infiltrates the entire bonding area through capillary action at a high temperature. Simultaneously, Si and C undergo a chemical reaction to form a stable SiC joint. The primary reaction at the interface can be represented as follows.

This reaction is highly temperature-dependent, with significant reaction rates achieved at temperatures above 1500 °C. The formation of the SiC layer at the interface is critical for joint performance as it prevents further Si infiltration. Factors such as the Si-to-C ratio, reaction time, and substrate surface roughness play essential roles in determining the efficiency and quality of the reaction.

Typical grain boundaries of the joint are generally Si/SiC, Si/Si, and SiC/SiC. Si-C reaction bonding is usually performed at high temperatures above 1500 °C, which helps Si react with C and form SiC. Sung et al. [17] used paper as a carbon source to keep it warm for 10~60 min at 1480 °C and realized the bonding of SiC through pressureless sintering. Luo et al. [97] also used reaction bonding to obtain the highest bending strength of 439 MPa.

The Si-C reaction bonding process involves the interaction between silicon (Si) and carbon (C) at high temperatures, typically above 1200 °C, forming silicon carbide (SiC) as the primary product. This reaction is temperature-dependent, with higher temperatures increasing the diffusion rates of Si and C atoms at the interface, promoting the formation of SiC. For effective bonding, the surfaces of Si and C must be clean, with surface activation methods like plasma treatment often used to prepare the surfaces. The presence of reactive gases (such as hydrogen or oxygen) or additives can influence the bonding by facilitating the formation of intermediate phases like silicon oxycarbide (SiOC) or silicon carbonitride (SiCN), which may affect the mechanical properties of the bond. The bonding strength is largely determined by the quality of the SiC interface, which is strong due to covalent bonding. However, thermal expansion mismatch between silicon and carbon poses challenges, potentially generating stress at the interface under thermal cycling. Overall, the Si-C bonding process is complex, involving multiple factors such as temperature, surface conditions, and additives, all of which impact the bonding strength and durability of the joint.

However, Si-C in the bonding layer cannot be completely consumed in the actual bonding reaction process, so residual Si often appears in the bonding layer, and the joint is often unsuitable for high-temperature application due to residual Si. There is inevitably free silicon in Si-C reaction bonding. To expand the application of Si-C reaction bonding in nuclear reactors, it is necessary to reduce the content of free silicon in the joint to reduce the anisotropic expansion of free silicon after neutron irradiation and reduce the strength of the joint [82]. Table 8 summarizes the bonding process and mechanical property evaluation of Si-C-reaction-bonding SiC and SiC matrix composites in recent years.

## 3. Conclusions and Outlook

Silicon carbide (SiC) materials have emerged as indispensable components in aerospace and nuclear applications due to their exceptional thermal stability, mechanical robustness, and resistance to harsh environments. However, their inherent brittleness and poor machinability pose significant challenges in fabricating complex geometries, making the development of advanced joining technologies a critical area of research.

Currently, SiC bonding techniques can be broadly categorized into pressure-assisted and pressureless methods. Pressure-assisted approaches—such as direct bonding, solid-state diffusion, and NITE phase bonding—typically yield joints with superior mechanical strength and density. Yet, these methods often require high temperatures and pressures, which can damage substrates and increase costs, limiting their scalability. In contrast, pressureless techniques like glass-ceramic bonding, polymer precursor bonding, and metal brazing offer cost-effective and industrially viable solutions, though they may sacrifice joint integrity under extreme conditions.

Looking ahead, several key directions warrant attention to advance SiC joining technologies:

1. Extreme Environment Performance: Future research must prioritize bonding solutions capable of withstanding high radiation, thermal cycling, and corrosive environments, particularly for nuclear and aerospace applications.
2. Process Optimization: Developing low-temperature and low-pressure bonding methods will be essential to minimize residual stresses and substrate degradation while maintaining joint reliability.
3. Hybrid Bonding Strategies: Combining the strengths of different techniques—such as integrating NITE phase bonding with Si–C reaction bonding—could unlock new possibilities for high-performance joints.
4. Computational and Data-Driven Approaches: Incorporating numerical simulations and machine learning could accelerate the optimization of bonding parameters, enabling the predictive modeling of joint behavior under operational stresses.
5. Material and Interface Engineering: Refining filler materials, surface treatments, and interfacial designs will be crucial to enhance joint durability and compatibility with base materials.
6. Real-World Validation: The systematic evaluation of bonded joints under practical operating conditions is needed to bridge the gap between laboratory research and industrial deployment.

By addressing these challenges, the field can unlock the full potential of SiC composites, enabling their broader adoption in next-generation high-temperature and high-stress applications. The integration of innovative bonding strategies with advanced computational tools promises to revolutionize the design and manufacturing of SiC-based components, paving the way for more efficient and reliable systems in aerospace, nuclear energy, and beyond.

## Figures and Tables

**Table 1:** Evaluation of bonding process and mechanical properties of SiC and SiC_f_/SiC composite bonding without bonding layers.

Bonding Base Material	Bonding Method	Bonding Condition	Joint Strength	Source
SiC-SiC	Electric-Current-Assisted Bonding	1750 °C, 10 min 2160 °C, 1 min	/	[26]
SiC-SiC	Spark Plasma Sintering (SPS)	1900 °C, 5 min, 60 MPa, SiC Surface Polishing	Bending strength 193 ± 21 MPa	[24]
		2000 °C, 5 min, 60 MPa, SiC Unpolished	Bending strength 68 ± 9 MPa	
SiC-SiC	DC-field-assisted Sintering	400 °C, 30 min, 2 MPa, Air	/	[11]
SiC-SiC	Hot pressing Sintering	1465 °C, 30–45 min, 0.23 MPa	/	[37]
SiCf/SiC (Hastelloy X)	Hot pressing Sintering	900 °C/1000 °C, 1 h, 200 MPa	/	[38]
SiCf/SiC (Incoloy 909)	Hot pressing Sintering	900 °C/1000 °C, 1 h, 200 MPa	/	[38]

**Table 2:** The bonding process and mechanical property evaluation of metal-solid-diffusion-bonded SiC and SiC matrix composites.

Bonding Base Material	Bonding Layer	Bonding Condition	Bonding Method	Joint Strength	Source
Cf/SiC-Cf/SiC (3D)	Ni-Ti-Nb-Ti-Ni	Spark Plasma Sintering (SPS)	1400 °C, 5 min, Vacuum, 50 MPa	Shear strength 108 ± 5 MPa	[46]
SiC-SiC	Ti-CoFeCrNiCu-Ti	Spark Plasma Sintering (SPS)	1200 °C, 15 min, Ar, 50 MPa	Bend strength 72 ± 2 MPa	[47]
SiC-SiC	Ta-5W (100 μm)	Spark Plasma Sintering (SPS)	1600 °C, 5 min, Ar, 30 MPa	Shear strength 122 ± 15 MPa	[48]
Cf/SiC-Cf/SiC (3D)	Ti-Nb-Ti	Spark Plasma Sintering (SPS)	1200 °C, 5 min, 30 MPa	Shear strength 61 ± 6 MPa	[40]
SiC-SiC	Ti	Spark Plasma Sintering (SPS)	1400 °C,10 min, Ar, 30 MPa	Shear strength 109.3 ± 4.5 MPa	[8]
SiC-SiC	Ti-Re-Ti	Hot Pressing Sintering	1400–1600 °C, 2 h, Ar, 25 MPa	/	[42]
SiC-SiC	Ti	Hot Pressing Sintering	1400 °C,1 h, Vacuum, 7 MPa	Shear strength 67 MPa	[49]
SiC-SiC	Mo	Hot Pressing Sintering	1400 °C, 1 h, Vacuum, 7 MPa	Shear strength 76 MPa	[49]
SiCf/SiC-SiCf/SiC	Ta	Pressureless Sintering	1500 °C, 5 h, vacuum	Shear strength 13.86 MPa	[50]
SiC-SiC	ZrSi_2_-SiC	Spark Plasma Sintering (SPS)	1600 °C, 30 min, 30 MPa	Shear strength 168.1 ± 12.6 MPa	[51]
SiC-SiC	Y_2_O_3_-ZrO_2_-Al_2_O_3_	Spark Plasma Sintering (SPS)	1600–1800 °C, 40 MPa	Bend strength 107.3 MPa	[52]
Cf/SiC-Cf/SiC	Y_3_Si_2_C_2_	Spark Plasma Sintering (SPS)	1600 °C, 10 min	Shear strength 17.2 ± 2.9 MPa	[53]
Cf/SiC-Cf/SiC	Pr_3_Si_2_C_2_	Spark Plasma Sintering (SPS)	1000–1500 °C, 30 s, 30 MPa, Vacuum	Shear strength 17.6 ± 3 MPa	[54]
SiC-SiC	W	Hot Pressing Sintering	1700–1900 °C, 10–120 min, Ar	Shear strength 90 MPa	[55]

**Table 3:** Bonding process and mechanical property evaluation of NITE-bonded SiC and SiC matrix composites.

Bonding Base Material	Bonding Layer	Bonding Method	Bonding Condition	Joint Strength	Source
SiC-SiC	Al_2_O_3_+CeO_2_+SiC	Spark Plasma Sintering (SPS)	1700 °C, 10 min, 20 MPa, Ar	Shear strength 163.9 MPa	[60]
SiC-SiC	Al_2_O_3_+Y_2_O_3_+SiC	Spark Plasma Sintering (SPS)	1700 °C, 10 min, 20 MPa, Ar	Shear strength 115 MPa	[60]
SiC-SiC	SiO_2_+Al_2_O_3_+Y_2_O_3_+SiC	Spark Plasma Sintering (SPS)	1700 °C, 10 min, 20 MPa, Ar	Shear strength 104.2 MPa	[60]
SiC-SiC	Al_2_O_3_+Ho_2_O_3_+SiC	Spark Plasma Sintering (SPS)	1700 °C, 10 min, 20 MPa, Ar	Shear strength 157.8 MPa	[57]
SiC-SiC	Al_2_O_3_+Y_2_O_3_+MgO+CaO+SiC	Spark Plasma Sintering (SPS)	1650 °C, 30 MPa, Vac	Shear strength 69.5 ± 8.9 MPa	[9]
SiC-SiC	SiO_2_+Al_2_O_3_+Y_2_O_3_+SiC	Hot Pressing Sintering	1800 °C, 20 MPa, 1 h	Tensile strength 249 MPa	[56]
SiC-SiC	SiC+AlN+Y_2_O_3_	Vacuum Sinter	1750 °C, 2 h, Ar	Bend strength 320.5 ± 37.6 MPa	[10]

**Table 4:** Bonding process and mechanical performance evaluation of MAX phase bonded SiC and SiC matrix composites.

Bonding Base Material	Bonding Layer	Bonding Method	Bonding Condition	Joint Strength	Source
SiC-SiC	Ti_3_SiC_2_	Hot Pressing Sintering	1900 °C, 3.5 MPa, 5 h, Ar	Bend strength 230.55 ± 18.96 MPa	[13]
SiC-SiC	Ti_3_AlC_2_	Hot Pressing Sintering	1900 °C, 3.5 MPa, 5 h, Ar	Bend strength 298.34 ± 9.38 MPa	[13]
Cf/SiC-Cf/SiC	Ti_3_SiC_2_	Hot Pressing Sintering	1200–1600 °C, 20–40 MPa, 30 min	Bend strength 110.4 MPa	[61]
SiCf/SiC-SiCf/SiC	Ti_3_SiC_2_	Spark Plasma Sintering (SPS)	1300 °C, 50 MPa, 5 min, Vac	Shear strength 18.3 ± 5.8 MPa	[62]
SiCf/SiC-SiCf/SiC	Ti_3_SiC_2_+SiC	Hot Pressing Sintering	1400 °C/1500 °C, 3.5 MPa, 2 h, Ar	Bend strength 198 MPa	[12]
SiC-SiC	Ti_3_SiC_2_	Spark Plasma Sintering (SPS)	1300 °C, 50 MPa, 5 min, Vac	Bend strength 220.3 ± 3.2 MPa	[63]

**Table 5:** Bonding process and mechanical performance evaluation of glass-ceramic-bonded SiC and SiC composites.

Bonding Baes Material	Bonding Layer	Bonding Method	Bonding Condition	Joint Strength	Source
SiC-SiC	CaO-Al_2_O_3_-MgO-TiO_2_-SiO_2_	Pressureless Sintering	1400 °C, 15 min, Ar	Shear strength 69.3 ± 9.9 MPa	[65]
SiC-SiC	CaO-Li_2_O-Al_2_O_3_-SiO_2_	Pressureless Sintering	1240 °C, 10 min, Ar	Shear strength 127 MPa	[66]
SiC-SiC	CaO-Al_2_O_3_-SiO_2_-Li_2_O	Pressureless Sintering	1260 °C, 10 min, Ar	/	[67]
SiC-SiC	CaO-Al_2_O_3_	Pressureless Sintering	1480 °C, 10 min, Ar	/	[68]
SiCf/SiC-SiCf/SiC	CaO-Y_2_O_3_-Al_2_O_3_-SiO_2_	Pressureless Sintering	1400 °C, 30 min, Ar	Shear strength 57.1 ± 6 MPa	[69]
SiC-SiC	MgO-Al_2_O_3_-SiO_2_	Pressureless Sintering	1450–1600 °C, 2 h, Ar	Four-point bending strength 286 ± 40 MPa	[70]
SiCf/SiC-SiCf/SiC	Y_2_O_3_-Al_2_O_3_-SiO_2_	Pressureless Sintering	1450 °C, 20 min, Ar	Shear strength 61 ± 12 MPa	[71]
SiC-SiC	CaO-Al_2_O_3_-SiO_2_	Pressureless Sintering	1450 °C, 10 min, Ar	Shear strength 86 MPa	[72]

**Table 6:** Bonding process and mechanical performance evaluation of polymer-precursor-bonded SiC and SiC matrix composites.

Bonding Base Material	Bonding Layer	Bonding Method	Bonding Condition	Joint Strength	Source
SiC-SiC	(-SiHCH_3_-CH_2_-)n(PCS)	Pressureless Sintering	1500 °C, 2 h, Ar	Shear strength 105.8 ± 10.4 MPa	[15]
SiC-SiC	Polysiloxane	Pressureless Sintering	1200 °C, 1 h, N2	Bend strength 197 MPa	[74]
SiC-SiC	Polymethylsiloxane, epoxy resin	Pressureless Sintering	1100 °C, 1 h, air	Shear strength 37.28 ± 1.33 MPa	[75]
SiC-SiC	PMS, D4Vi	Pressureless Sintering	1000 °C, 2 h, N2	Shear strength 34.5 ± 4.6 MPa	[78]
SiC-SiC	Polycarbosilane (PCS), ferrocene	Pressureless Sintering	Step1: 1300 °C, 2 h, Ar; step2: 1600 °C, 1 h	/	[76]

**Table 7:** The bonding process and mechanical performance evaluation of metal brazing, bonded for SiC and SiC composites.

Bonding Base Material	Bonding Layer	Bonding Method	Bonding Condition	Joint Strength	Source
SiC-SiC	(Ni-56Si)-Mo-(Ni-56Si)	Pressureless Sintering	1350 °C, 10 min, vacuum	Shear strength 41 MPa	[83]
Cf/SiC-Cf/SiC (2D)	Cu-18Au-32Pd-(6–10) V	Pressureless Sintering	1120 °C, 10 min, vacuum	Bend strength 135 MPa	[84]
SiC-SiC	Ni-28Mo	Pressureless Sintering	1300 °C, 40 min, vacuum	Bend strength 174 ± 11 MPa	[85]
SiCf/SiC-SiCf/SiC	Ti16Si84	Pressureless Sintering	1410 °C, 10 min, Ar	Shear strength 42.5 MPa	[18]
SiC-SiC	Al_3_Ti-Ti-Al_3_Ti	Pressureless Sintering	1500 °C, 10 min, Ar	Shear strength 89 MPa	[80]
SiC-SiC	Si(powder)- Ti (Foil)- Si(powder)	Pressureless Sintering	1500 °C, 1 h, vacuum	Shear strength 76.2 ± 19.6 MPa	[86]
SiC-SiC	Sn-Ti	Pressureless Sintering	950 °C, 10 min, vacuum	Shear strength 27–32 MPa	[87]
SiC-SiC	Si-24Ti-3 Carbon nanotube	Pressureless Sintering	1380 °C, 20 min, Ar	Shear strength 88.5 MPa	[88]
SiC-SiC	Ni Foil	Pressureless Sintering	1245 °C, 60 min, vacuum	Shear strength 29.43 MPa	[39]
SiC-SiC	Si-Al	Pressureless Sintering	900 °C, 30 min, vacuum	Bend strength 279 ± 13 MPa	[89]
SiCf/SiC- SiCf/SiC	Mo-Si	Pressureless Sintering	1450 °C, 1–10 min, Ar	Shear strength 7~10 MPa	[90]
Cf/SiC-GH3536	Cu-Ti-WC	Pressureless Sintering	1140 °C, 20 min, vacuum	Shear strength 74.3 MPa	[91]
SiCf/SiC-Al0.3CoCrFeNi	Cu-Ti	Pressureless Sintering	1125 °C, 10 min, vacuum	Shear strength 59 MPa	[92]
SiCf/SiC-GH536	CoFeNiCrCu	Pressureless Sintering	1160 °C, 60 min, vacuum	Shear strength 86.07 ± 4.5 MPa	[93]
SiCf/SiC-GH536	CoFeCrNiCuTi	Pressureless Sintering	1160 °C, 60 min, vacuum	Shear strength 98.23 ± 5.12 MPa	[94]
SiCf/SiC-GH536	CoFeNiCrMnNb-MoNiSi-CoFeNiCrMnNb	Pressureless Sintering	1200 °C, 20 min, vacuum	Shear strength 98.1 MPa	[95]
SiCf/SiC-GH536	CoFeNiCrMnNb	Pressureless Sintering	1200 °C, 20 min, vacuum	Shear strength 89.7 MPa	[96]
SiC-Mo	Nb_0.74_CoCrFeNi_2_	Pressureless Sintering	1300 °C, 15 min, vacuum	Shear strength 62 MPa	[81]

**Table 8:** Bonding process and mechanical evaluation performance of Si-C-reaction-bonding SiC and SiC composites.

Bonding Base Material	Bonding Layer	Bonding Method	Bonding Condition	Joint Strength	Source
SiC-SiC	SiC, C	Pressureless Sintering	1450 °C, 30 min, vac	Bend strength 439 ± 31 MPa	[97]
SiC-SiC	SiC, C	Pressureless Sintering	1600 °C, 30 min, vac	Bend strength 308 ± 27 MPa	[98]
Cf/SiC- Cf/SiC	SiC, C	Pressureless Sintering	1600 °C, vac	Bend strength 203 ± 24 MPa	[99]
SiC-SiC	SiC, C	Pressureless Sintering	1450 °C~1550 °C, 10~60 min, vac	Bend strength 243~246 MPa	[17]

## Data Availability

The datasets generated and/or analyzed during the current study are available upon reasonable request from the corresponding author. All relevant data, codes, and materials have been included in the manuscript. The research data referred to in this study are appropriately cited in the references section.

## Nomenclature

NITE	Nano-Infiltration and Transient Eutectic
CVl	Chemical Vapor Infiltration
CET	Coefficient of thermal expansion
XRD	X-ray diffraction
FEA	Finite element analysis
SPS	Spark Plasma Sintering
ECAJ	Electric-Current-Assisted Bonding
CVD	Chemical Vapor Deposition
PCS	Polycarbosilane
EDS	Energy-Dispersive Spectroscopy
SEM	Scanning electron microscopy
